# Parental perceptions of the importance of pediatric out-of-hospital cardiopulmonary resuscitation for the survival rate in Saudi Arabia: a cross-sectional survey

**DOI:** 10.1186/s12245-023-00564-3

**Published:** 2023-11-03

**Authors:** Nouf S. Almutairi, Nesrin A. Alharthy, AlAnoud M. Almaziad, AlJazi T. Alsalloum, Rozanna A. AlHarbi, Shamayel A. Almulhem, Amal Yousif, Fatmah Othman

**Affiliations:** 1 College of Medicine Department, King Saud Bin Abdulaziz University for Health Sciences, Riyadh, Saudi Arabia; 2 College of Applied Medical Sciences-Riyadh, King Saud Bin Abdulaziz University for Health Sciences, PO Box 22490, 11426 Riyadh, Saudi Arabia; 3 Pediatric Emergency Department, King Abdulaziz Medical City Ministry of National Guard Health Affairs, PO Box 22490, 11426 Riyadh, Saudi Arabia; 4 King Abdullah International Medical Research Center, Riyadh, Saudi Arabia; 5 College of Public Health and Health Informatics, King Saud Bin Abdulaziz University for Health Sciences, Riyadh, Saudi Arabia

**Keywords:** Pediatric cardiac arrest, Bystander cardiopulmonary resuscitation, Out-of-hospital cardiopulmonary resuscitation, Basic life support, Awareness, Saudi Arabia, Parents

## Abstract

**Background:**

Pediatric out-of-hospital cardiac arrest is associated with high morbidity and mortality rates. Cardiopulmonary resuscitation (CPR), the practice of chest compressions combined with rescue breathing, is crucial for the success of out-of-hospital resuscitation after sudden cardiac arrest. Thus, imparting the requisite knowledge and skills to parents/caregivers can significantly enhance survival rates. This study investigated parental awareness of the impact of out-of-hospital pediatric CPR on survival rates in Saudi Arabia.

**Methods:**

This cross-sectional study was conducted using an online questionnaire administered to Saudi parents from all regions of the Kingdom of Saudi Arabia. Data were collected using the convenience sampling method, as the questionnaire was distributed via social media platforms. The questionnaire consisted of five parts: (1) demographic data, (2) questions about parents’ perception of basic life support (BLS), (3) evaluation of parents’ knowledge of the impact of prehospital CPR on survival rates, (4) measurement of parents’ competency in performing pediatric CPR, and (5) assessment of whether parents’ confidence was affected by prior training. Statistical analyses were conducted using the chi-squared test or Fisher’s exact test, and the t-test was used to compare the mean scores of the groups of parents with medical and non-medical professional backgrounds.

**Results:**

A total of 1,065 individuals responded to the survey. The respondents’ mean age was 41 ± 0.2 years and 46.5% were men. We found that 73.9% of respondents had no prior experience with BLS, 87% had never taken a BLS course, and 61% did not know where to find one. The majority of participants agreed that bystander CPR contributes to overall survival rates, and 77% agreed to the importance of BLS training. Medical professionals showed a higher percentage of agreement on the importance of BLS than those from non-medical backgrounds (90% vs. 76%, *p* = 0.036), especially parents of high-risk children.

**Conclusion:**

This study showed evidence of interest in CPR and BLS training in Saudi parents, despite the low levels of knowledge regarding BLS training.

## Introduction

Cardiac arrest refers to the abrupt loss of cardiac function in the event of malfunctioning of the electrical system of the heart; consequently, the heart stops beating properly. Cardiac arrest can result in death if appropriate measures are not implemented immediately [1]. According to the American Heart Association, the pediatric cardiac chain of survival includes prevention, early cardiopulmonary resuscitation (CPR), seeking help, quick implementation of advanced pediatric life support, and aggressive post-resuscitation care [2]. The purpose of CPR is to maintain active blood flow through chest compressions with mouth-to-mouth artificial ventilation, and, if possible, to manually preserve spontaneous circulation of oxygenated blood [1–3]. Moreover, the availability and use of a defibrillation shock device, implementation of early advanced life support by trained personnel, and comprehensive post-cardiac arrest care increase the chances of survival and improve outcomes [4].

Immediate resuscitation after cardiac arrest is essential to increase the survival rate [2]. Studies have reported that continuous chest compressions alone during CPR are undoubtedly preferred over no CPR [5]. The probability of survival after cardiac arrest in pediatric patients with early CPR initiation and defibrillator use during the first 4 min has been reported to be 43% [6]. Furthermore, every minute without CPR reduces the chance of survival by 7–10% [4–6]. Studies on pediatric in-hospital and out-of-hospital cardiopulmonary arrest (CPA) events have found that the hospital survival rate increases with the implementation of the chain of survival [7]. This finding highlights the importance of mastering CPR skills and their early application [7]. Despite the tremendous effect of CPR on survival rates, a study conducted from 1989–1995 on all out-of-hospital and emergency department (ED) cardiac arrests treated at a tertiary care hospital in Riyadh found that only 3% of patients received bystander CPR [8].

The lack of awareness and training in CPR among parents constitutes a critical international predicament [9, 10]. According to a study conducted in Japan, only 20.8% of female parents and 20.4% of male parents knew how to perform CPR [9]. Moreover, Concepción's study reported limited awareness and lack of official CPR training among parents in Madrid [11]. Few studies have examined parental awareness in Saudi Arabia at the national level. A study conducted in Al-Khobar found that 67.2% of 753 participants had no knowledge of CPR despite having at least one child with a disability [12]. Furthermore, Alenezi e al. reported that most mothers in the Dammam region lacked knowledge of CPR, but exhibited the incentive to learn it. Another study conducted in Jeddah found that, although participants had a significantly low level of CPR training, they expressed concerns about making mistakes during CPR [13, 14]. Few studies have investigated parents' knowledge of children's survival rates following out-of-hospital CPR in Saudi Arabia. Therefore, this study aimed to include all administrative regions of Saudi Arabia and analyze parental awareness of the impact of out-of-hospital CPR on survival rates.

## Methods

This cross-sectional study entailed a survey conducted between June and July 2021. A non-probability convenience sampling technique was employed to select respondents, yielding in a total sample size of 1065. The fourth survey conducted by the Directorate-General for Statistics in Saudi Arabia included 4,585,017 married Saudi women with children [15] and was considered the last one in the five-year survey. According to Raosoft's sample size calculator, 385 participants were required, with a confidence level of 95%, an error rate of 5%, and an average response rate of 50%.

Experts reviewed the existing literature and developed a questionnaire on pediatric emergency medicine and the BLS course. First, the questions were reviewed and modified, followed by development of the questionnaire in English, translation into Arabic, and translation back into English. A pilot test was performed with 12 individuals to assess the reliability and validity (clarity and logical reasoning) of the questionnaire. Before data collection, the survey was modified to clarify ambiguous questions. Cronbach's alpha coefficient for the reliability of the scale was 0.63.

### Data management and analysis plan

The data were coded, edited, entered into Microsoft Excel, and analyzed using the Statistical Package for Social Sciences (SPSS) version 22. Categorical variables (sex, nationality, marital status, residential area, education, and profession) were reported as frequencies and percentages.

Similarly, variables including the level of knowledge and level of confidence regarding CPR were measured using a five-point Likert scale and presented as frequencies and percentages. The continuous variable (age) was presented as the maximum, minimum, and mean ± standard deviation. Parents' competency was also scored and presented as the mean and standard deviation.

Furthermore, the chi-squared or Fisher's exact test was used to evaluate the parents' perceptions of CPR training and their demographic information if there were fewer than five participants. Fisher's exact tests were also used to determine the effect of previous CPR training on parental confidence.

The secondary objective, i.e., CPR training of parents of medically high-risk children and CPR training of those with healthy children, was compared using the chi-squared or Fisher's exact test. The mean scores of parents with and without medical backgrounds were compared using t-tests. Statistical significance was set at *p* ≤ 0.05.

## Results

### Description of the study population

An online survey was conducted with 1065 individuals. Of the 1065 participants, 46.5% were men and 53.5% were women. The respondents’ age distribution was as follows: 18–30 years, 8.54%; 31–40 years, 41.50%; 41–50 years, 37.56%; and above 50 years, 12.4% (*n* = 132). Riyadh was the most common region of origin (50.5%), followed by Al-Qaseem (16.3%). There were 95.9% married respondents and the rest were divorced, widowed, or caregivers. Forty-six (51.3%) respondents had a bachelor’s degree, and 20.8% had a high school diploma. As shown in Table 2, 29% of respondents earned between 4001 and 10,000 Riyals. Most respondents (92%) were not medical professionals, and only 8% had a medical background (Table 1).

**Table 1:** Sociodemographic description of the study population

Characteristic	Total (*n* = 1065)	Percentage (%)
***Gender***		
Male	495	46.5
Female	570	53.5
***Age (mean, SD)***	41 (0.26)	
***Age Groups***		
18–30 years	91	8.54
31–40 years	442	41.50
41–50 years	400	37.56
51 years and above	132	12.4
***City of Residence***		
Riyadh	538	50.5
Al-Qaseem	174	16.3
Hail	31	2.9
Makkah	91	8.5
Al-Madina	45	4.2
Eastern Region	87	8.2
Aseer	27	2.5
Jazan	5	0.5
Tabouk	28	2.6
Najran	15	1.4
Al-Jouf	4.0	0.4
Al-Baha	11	1.0
***Marital Status***		
Married	1021	95.9
Single	44	4.1
***Education***		
Below high school	81	7.6
High school certificate	221	20.8
Technical and further education	133	12.5
Bachelor’s degree	546	51.3
Postgraduate	83	7.8
Others	1.0	0.1
***Socioeconomic Status (Income in Riyals)***		
< 4000	220	20.7
4001–10000	310	29.1
10,001–15000	297	27.9
15,001–20000	141	13.2
> 20,000	97	9.1
***Profession***		
Medical	85	8.0
Nonmedical	980	92.0

Approximately 55% of the participants had children aged 1–3 years, 37% had children aged 4–6 years, and only 7 participants had children aged 10 years or above; 72 (6.8%) participants reported that they had children with disabilities or chronic illnesses (Table 1).

### Parents’ perceptions, knowledge and competency of pediatric CPR training

BLS was unfamiliar to 74.9% of participants. Although 26.1% of respondents had previously heard of BLS, only 12.6% had taken a BLS course. The majority (53.72%) of BLS-trained participants reported they had not participated in CPR training in over two years. Another 61.3% of participants did not know where to acquire BLS training. Furthermore, 77.2% agreed that all parents should receive BLS training (Table 2). Parents lacked knowledge about pediatric CPR, including items 2, 4, 6, and 10, from the questionnaire. The location of the pulse point was unknown to 75.9% of participants. Furthermore, 66.4% of participants reported inadequate knowledge of CPR (Table 3).

**Table 2:** Parents’ perceptions of pediatric cardiopulmonary resuscitation

Item	Option	Number and Frequency (%)
1. Do you have previous information about BLS?	No	787 (73.9)
	Yes	278 (26.1)
2. Have you ever taken a BLS course?	No	931 (87.4)
	Yes	134 (12.6)
3. If yes, time since previous CPR training	< 6 months	25 (18.66)
	7 to 12 months	17 (12.69)
	13 to 24 months	20 (14.93)
	> 2 years	71 (53.72)
4. If no, reasons for no previous training	Little interest	188 (17.7)
	Little time	172 (16.2)
	Do not know where to get it	653 (61.3)
	Costs too much	101 (9.5)
	Others	37 (3.5)
5. Do you think all parents should receive BLS training?	Strongly Disagree	31 (2.9)
	Disagree	10 (0.9)
	Neutral	104 (9.8)
	Agree	98 (9.2)
	Strongly Agree	822 (77.2)

*BLS* basic life support, *CPR* cardiopulmonary resuscitation

**Table 3:** Parents’ knowledge and competency regarding pediatric cardiopulmonary resuscitation

Item	Correct N (%)	Incorrect N (%)
1. What is the best thing that you can do when you find an infant/child unconscious or unresponsive?	613 (57.6)	452 (42.4)
2. The location to check for a pulse in an infant:	257 (24.1)	808 (75.9)
3. The location to check for a pulse in children older than 1 year:	625 (58.7)	440 (41.3)
4. How do you confirm that a child is not breathing when he or she is unconscious?	334 (31.4)	731 (68.6)
5. If you witness an unconscious child and there is a pulse, but he or she is not breathing, what should you do?	702 (65.9)	363 (34.1)
6. Hand placement during chest compressions:	475 (44.6)	590 (55.4)
7. What is the ratio of chest compression to effective breaths when performing CPR on a child?	696 (65.4)	369 (34.6)
8. Depth of chest compressions:	583 (54.7)	482 (45.3)
9. If you witness a conscious child unable to breathe and choking on a piece of toy he or she has put into his or her mouth, what should you do?	808 (75.9)	257 (24.1)
10. How should you give chest compression to a child older than 1 year?	358 (33.6)	707 (66.4)

### Association between the mean competency score and participants’ demographic characteristics of

Parents with a medical background were compared to those without such a background. The mean ± SD competency score was 5.12 ± 0.05 (Table 4). Male participants scored higher (5.14 ± 1.7) than female participants [(5.09 ± 1.7), t (1063) = 0.408, *p* = (0.683)]. Similarly, participants with a medical background scored higher (5.73 ± 1.7) than those with a non-medical background [(5.07 ± 1.7), t (1063) = 3.43, *p* = 0.149)]. Contrary to expectations, there were no differences in the mean scores of participants with and without chronically ill children (Table 4).

**Table 4:** Association between the mean competency score and participants’ demographic characteristics

Variable	Mean	Standard Deviation	*P* value
***Overall mean***	5.12	0.05	
***Gender***			0.683
Male	5.14	1.73	
Female	5.09	1.71	
***Background***			0.149
Medical	5.73	1.75	t = 3.4323
Non-medical	5.07	1.71	
***Chronic conditions***			0.019
No	5.11	1.72	t = 0.3179
Yes	5.18	1.78	

### Parents’ knowledge about the effect of prehospital CPR on the survival rate

About 73.5% of participants reported that bystander CPR contributed to overall survival (mean score = 4.49 ± 1.0). Participants aged 31 to 40 years had the highest knowledge score (4.53 ± 0.98). Participants with higher education levels were more aware of the impact of prehospital CPR. The mean score of parents with postgraduate degrees was higher than that of parents with a high school diploma (mean score = 4.67 ± 0.75 vs. mean score = 4.38 ± 1.05, *p* = 0.798). Additionally, 93.7% of respondents indicated that BLS training should be provided to parents of high-risk children.

### Effect of previous CPR training on parents' confidence levels

Previous CPR training influenced parents' confidence. Most participants believed that they lacked knowledge on BLS (29.6% strongly disagreed, 29.6% disagreed) and lacked confidence in their CPR abilities (34% strongly unconfident, 24.1% underconfident). Further, 85.3% of participants had never encountered a situation requiring CPR. More than 90% of respondents felt that additional training was required (Table 5).

**Table 5:** Parents' knowledge score of prehospital the impact of cardiopulmonary resuscitation and their confidence levels after previous training

Item	Frequency (%)
***Do you think that bystander CPR could impact the survival rate?***	
Strongly Disagree	41 (3.8)
Disagree	17 (1.6)
Neutral	100 (9.4)
Agree	124 (11.6)
Strongly Agree	783 (73.5)
***Parents' knowledge score based on age***	**(Mean, SD)**
18–30 years	4.49 (0.91)
31–40 years	4.53 (0.98)
41–50 years	4.46 (1.05)
51 and above	4.52 (0.96)
***Parents' knowledge score based on education***	**(Mean, SD)**
Below high school	4.33 (1.20)
High school certificate	4.38 (1.05)
Technical and further education	4.43 (1.05)
Bachelor’s degree	4.55 (0.96)
Postgraduate	4.67 (0.75)
***Do you think parents with medically high-risk children who are prone to cardiac arrest should receive BLS training?***	**Frequency (%)**
Strongly Disagree	23 (2.2)
Disagree	03 (0.3)
Neutral	22 (2.1)
Agree	19 (1.8)
Strongly Agree	998 (93.7)
***Score based on age***	**(Mean, SD)**
18–30 years	4.88 (0.55)
31–40 years	4.81 (0.76)
41–50 years	4.87 (0.59)
51 and above	4.88 (0.63)
***Score based on education***	**(Mean, SD)**
Below high school	4.85 (0.69)
High school certificate	4.81 (0.69)
Technical and further education	4.92 (0.41)
Bachelor’s degree	4.83 (0.74)
Postgraduate	4.92 (0.46)
Others	5.0 (0.0)
***Do you feel your knowledge about BLS is sufficient?***	
Strongly Disagree	315 (29.6)
Disagree	315 (29.6)
Neutral	273 (25.6)
Agree	58 (5.4)
Strongly Agree	104 (9.8)
***Do you feel confident enough to perform CPR?***	
Strongly Unconfident	362 (34.0)
Unconfident	257 (24.1)
Neutral	253 (23.8)
Confident	95 (8.9)
Strongly Confident	98 (9.2)
***If you’ve previously taken a BLS course, did you feel more confident after the training?***	
Strongly Disagree	119 (11.2)
Disagree	41 (3.8)
Neutral	156 (14.6)
Agree	193 (18.1)
Strongly Agree	556 (52.2)
***Have you ever encountered a situation that requires CPR?***	
No	908 (85.3)
Yes	157 (14.7)
***Do you want more training?***	
No	106 (10)
Yes	959 (90)

*BLS* basic life support, *CPR* cardiopulmonary resuscitation

The results showed that 68.24% of the participants with a medical background had prior knowledge of BLS, while only 22.45% of non-medical participants had such knowledge (χ^2^: 82.84, *p* = 0.000). Only 8.67% of non-medical participants had attended a BLS course in the past, whereas 57.65% of participants with a medical background had taken a BLS course (χ^2^: 166.18, *p* < 0.001) (Table 6).

**Table 6:** Association between parents’ knowledge and their professional background

*Item*	Medical (*n* = 85)	Non-medical (*n* = 980)	Total (*n* = 1065)	*P* value*
***Do you have previous information about BLS?***				
No	27 (32.14%)	760 (77.55%)	787 (73.97%)	< 0.001
Yes	58 (68.24%)	220 (22.45%)	277 (26.03%)	
***Have you ever taken a BLS course?***				
No	36 (42.86%)	895 (91.33%)	931 (87.50%)	< 0.001
Yes	49 (57.65%)	85 (8.67%)	133 (12.50%)	
***Do you think all parents should receive BLS training?***				
Strongly Disagree	2 (2.35%)	29 (2.96%)	31 (2.91%)	0.036
Disagree	0 (0.0%)	10 (1.02%)	10 (0.94%)	
Neutral	4 (4.71%)	100 (10.20%)	104 (9.77%)	
Agree	2 (2.35%)	96 (9.80%)	98 (9.20%)	
Strongly Agree	77 (90.59%)	745 (76.02%)	822 (77.18%)	
***Do you think that bystander CPR could impact the survival rate?***				
Strongly Disagree	4 (4.71%)	37 (3.78%)	41 (3.85%)	0.83
Disagree	2 (2.35%)	15 (1.53%)	17 (1.60%)	
Neutral	9 (10.59%)	91 (9.29%)	100 (9.39%)	
Agree	10 (11.76%)	114 (11.63%)	124 (11.64%)	
Strongly Agree	60 (70.59%)	723 (73.78%)	783 (73.52%)	
***Do you think parents with medically high-risk children who are prone to have cardiac arrest should receive BLS training?***				
Strongly Disagree	2 (2.35%)	21 (2.14%)	23 (2.16%)	0.39
Disagree	0 (0.0%)	3 (0.31%)	3 (0.28%)	
Neutral	0 (0.0%)	22 (2.24%)	22 (2.07%)	
Agree	3 (11.76%)	16 (11.63%)	19 (11.64%)	
Strongly Agree	80 (94.12%)	918 (93.67%)	998 (93.71%)	

*BLS* basic life support, *CPR* cardiopulmonary resuscitation

^*^p values were obtained using the chi-squared test or Fisher's exact test as appropriate,

In addition, parents were asked about the level of importance of BLS courses. Approximately 90% of participants with a medical background believed that BLS training was essential for parents, while 76.02% of participants without a medical background concurred. Bystander CPR positively affected survival rates according to 75% and 73% of participants with and without a medical background, respectively (Fisher's exact: 0.83) (Table 6).

Additionally, 94.12% and 93.67% of participants with and without a medical background believed that the parents of medically high-risk children should receive BLS training, respectively (Fisher's exact: 0.39) (Table 6).

## Discussion

The current study investigated parental perceptions of the impact of out-hospital CPR on children's survival rates. The study found low levels of CPR knowledge among Saudi parents that varied with the demographic characteristics. However, parents were enthusiastic to learn CPR techniques. An overwhelming majority of parents acknowledged the universal importance of CPR education for parents of children with chronic illnesses.

Saudi parents who participated in this research reported low CPR knowledge. Most respondents had never taken a BLS course or heard of BLS. Among the minority (12.6%) who had received BLS training, 68% had received it over a year ago. This lack of awareness is consistent with that reported by other studies. For example, a study showed that only 20.6% of parents in Japan knew or received BLS training [9]. Other studies conducted in Madrid and New Zealand revealed poor BLS training awareness [11]. Moreover, this study also concurred with existing studies in Saudi Arabia that demonstrated a lack of awareness of CPR among 67.2% of parents, including parents of children with disabilities [12]. However, this finding was juxtaposed with a high level of interest in learning BLS and CPR.

According to most respondents, BLS training is necessary for parents. In exploratory studies, parents have expressed their desire for BLS training after the birth of their children to reduce their own anxiety and improve confidence [16]. These two findings indicate the necessity of exploring healthcare policy and decision-making in BLS training programs [16]. Studies have shown that BLS training significantly improves knowledge among untrained populations. It could positively impact the health assessment of patients who fall ill outside the hospital. Infant BLS training among parents has been shown to have clinically significant outcomes, including altering the outcomes of children with out-of-hospital CPA incidents [6]. Thus, engaging parents' interest in learning about CPR and other BLS training, especially for infants, has been associated with positive outcomes for infants at risk of CPA.

This study also provided insights into the demographic characteristics associated with BLS and CPR. The effect of bystander CPR on survival rates was related to BLS knowledge, participant age, and education level. The impact of bystander CPR on survival was more significant for young and educated respondents. Studies have almost unanimously reported that younger people are more likely to know health emergency practices than older people [17]. A Turkish study showed that a higher proportion of residents living in highly educated parts of the country had received BLS training and performed bystander CPR compared with those residing in low-education areas [18]. More recent studies have confirmed this finding by identifying age and education level as social determinants of health [19]. In this sense, these variables could also be interpreted as determining emergency health actions [20]. Assessment of participants' attitudes regarding the importance of parental BLS knowledge revealed similar levels of agreement, irrespective of age and education level. This implies the perceived importance of CPR and other BLS training among Saudi residents.

The near-universal interest in learning about CPR is a key finding of this research that is novel for the current population. Previous studies exploring parental attitudes regarding CPR and other BLS training reported parental fear of making mistakes and a lack of knowledge regarding CPR and other BLS training [12–14]. Alenezi et al. [13] reported maternal lack of knowledge on CPR, which incentivized education. However, the present research reveals new findings indicating interest in learning CPR and other BLS training among Saudi mothers and fathers. This finding is consistent with other studies demonstrating the value that parents attach to BLS training. It also demonstrates the Impact of BLS training on parental outcomes among at-risk children. Míguez-Navarro et al. [11] reported similarly low levels of parental knowledge of CPR and other BLS training, which indicated a strong need for BLS learning among parents. Universal interest among parents in learning CPR and other BLS methods implies that the demand for this training is already present. Policymakers should create systems designed to meet these needs among new parents and those with older children.

The study’s findings suggest that Saudi parents are interested in CPR and BLS training but lack access to it. Bystander CPR rates have risen due to public education programs [20]. Additionally, previous authors have recommended increasing the frequency of CPR training, in addition to the use of an automated electric defibrillator, which would improve bystanders willingness to perform CPR. Studies indicate that bystanders are more willing to provide CPR if they have received training in the past five years or attended at least three training sessions [21]. Saudi healthcare stakeholders can leverage the available research to formulate policies and programs that organize training sessions for new parents and parents of children living with disabilities. Such training will bridge the knowledge and practice gap among Saudi parents and significantly improve health outcomes for out-hospital medical emergencies.

Future studies should also address the limitations of this research, which originated from the sampling method. Convenience sampling creates representation bias. Half of the respondents in this research were from Riyadh, and 83.5% hailed from 4 of 13 regions in Saudi Arabia. This implies some under-representation in other regions. Future research should include participants from a broader geographical area to ensure broader regional inclusivity so that the results can be more generalizable.

## Conclusion

This study explored the attitudes of Saudi parents toward emergency BLS training. Despite the lack of knowledge about BLS training among this demographic, there is evidence of interest in CPR and BLS competence. The rate of parental CPR training in Saudi Arabia is about 20%, similar to developed and developing countries. Similarly, there are high levels of interest in learning CPR and BLS in parent groups internationally, regardless of age and education level. Age and education level predict BLS knowledge in Saudi Arabia, which could provide starting points for training programs. Since younger individuals are more likely to be parents to infants, this population may present an appropriate starting point for healthcare policymakers. This training is critical since it significantly improves long-term health outcomes for children experiencing CPA incidents or those at risk of adverse medical events, such as children with disabilities.

This study creates opportunities for research in other areas. As this study explored parental attitudes towards BLS training in Saudi Arabia's administrative regions, future research could explore the creation of novel and evidence-based BLS training or examine its effects on parent populations across the region.

## Data Availability

The data acquired during the current study are available from the corresponding author on reasonable request.

## Abbreviations

CPR: Cardiopulmonary resuscitation
BLS: Basic life support
CPA: Cardiopulmonary arrest
SPSS: Statistical Package for Social Sciences
